# Oribatid mites in different Mediterranean crop rotations fertilized with animal droppings

**DOI:** 10.1007/s10493-023-00812-4

**Published:** 2023-06-20

**Authors:** Àngela D. Bosch-Serra, M. Gabriela Molina, Elena González-Llinàs, Rosalia R. Boixadera-Bosch, Belén Martínez, Jordi Orobitg, Noemí Mateo-Marín, Francesc Domingo-Olivé

**Affiliations:** 1 Department of Chemistry, Physics and Environmental and Soil Sciences, University of Lleida, Av. Alcalde Rovira Roure 191, Lleida, E-25198 Spain; 2 Cátedras de Bioestadística I y II, Facultad de Ciencias Exactas Físicas y Naturales, Universidad Nacional de Córdoba, Vélez Sarsfield 299, Córdoba, Argentina; 3 Independent Scientist, Carrer Església 14, Puig-Reig, Barcelona, E-08692 Spain; 4 IRTA Mas Badia, Mas Badia Agricultural Experimental Station, E-17134, La Tallada d’Empordà, Girona, Catalonia Spain

**Keywords:** Acarina, Bioindication, Manure, Microarthropod, Slurry, Soil biota

## Abstract

Unsustainable soil management is one of the drivers of soil degradation, but impact assessment requires the development of indicators. Oribatids might be considered as early indicators of disturbances due to the stability of their community. The aim of this study was to investigate the feasibility of oribatids as bioindicators of sustainable agricultural practices. Under a dry Mediterranean climate, three fertilization experiments – two under a two-crop rotation system and one under maize monoculture and established 12 years earlier – were sampled 3× for oribatid identification during the last annual cropping cycle. The hypothesis was that different nutrient and crop managements affect the number of oribatid species and individuals present, and these parameters could be used as indicators of soil degradation. In total, 18 oribatid species were identified, and 1974 adult individuals were recovered. Maximum abundance was found prior to sowing. Pig slurry (PS) vs. control, and dairy cattle manure (CM) vs. mineral fertilization increased oribatid abundance. This increase was evident when the average applied rates with PS were ca. 2 Mg of organic matter (OM) ha^− 1^ yr^− 1^, or higher than ca. 4 Mg OM ha^− 1^ yr^− 1^ for CM. When the preceding crop was wheat and PS or CM were used, *Oribatula* (*Zygoribatula*) *excavata* (which reproduces sexually) predominated. In maize monoculture fertilized with CM, *Tectocepheus sarekensis* and *Acrotritia ardua americana* (which can reproduce through parthenogenesis) prevailed vs. *Oribatula*, which indicated a heavily disturbed soil. Under this specific Mediterranean environment, the predominance of certain parthenogenic oribatid species and the number of individuals provide advanced warning on soil degradation.

**Supplementary Information:**

The online version contains supplementary material available at 10.1007/s10493-023-00812-4.

## Introduction

Soil health is a worldwide issue (FAO and ITPS 2015). In the European Union, it has been stated that 60–70% of its soil ecosystems are unhealthy and suffering for continuous physical, chemical or biological degradation which hinders the harmonious development of soil services as nutrient or carbon cycles (European Commission 2021). In Mediterranean Europe, there is a great diversity of agricultural systems that are nowadays being questioned in terms of their sustainability (Migliorini et al. 2018). One of these systems is the agriculture devoted to cereals, which is usually linked to an important animal rearing activity (European Commission 2022), and where animal droppings are mainly reused as fertilizers. The current Common Agricultural Policy implements changes over agriculture management based on an eco-scheme concept which deals with different points, as the diversification of crop rotations and the maintenance of soil fertility (European Commission 2020). However, this common policy is facing the challenge of indicators to assess sustainable agricultural practices (Bouma et al. 2022).

Oribatid mites are a diverse group of terrestrial acarines, inhabiting the soil’s upper layers. The stability of community composition and their reproduction (sexually or through parthenogenesis) allow to consider them as early indicators of environmental changes due to human activities (van Straalen 1998; Mangová 2019). Nevertheless, there is a lack of studies of oribatids in agricultural fields. This research gap might be related to the fact that agricultural practices affect the amount of organic matter, the most important variable for detritivorous soil microarthropods that determines their organization (Hasegawa 2001). However, it has been described that oribatid mites also respond to crop and variety selection (Saowong et al. 2022a, b), in addition to responding to pesticide treatments (Behan-Pelletier 1999). In fact, pesticide applications have also been described as species diversity reducer in pine plantations (Hoy 1980). Cultivation may also reduce the presence and diversity of Oribatida (Coleman et al. 2018; Rockett 1986). Other authors have found that oribatid species are also sensitive to negative disturbances related to agricultural management such as over-fertilization (Bosch-Serra et al. 2014). There are some articles dealing with microarthropods and fertilization. In a wheat field, Zheng et al. (2019) described that soil mite community taxa were more directly responsive to changes in soil environmental parameters than to irrigation and nitrogen fertilizer management. In grasslands, Bolger and Curry (1984) and Sokołowska and Seniczak (2005) concluded that pig slurry or cattle liquid manure, applied at rates related to the nutrient requirements, did not affect microarthropod community or even increase the density of Oribatida. In addition, Graczyk et al. (2008) found that the use of cattle slurry manure (60–80 m^3^ ha^− 1^) significantly decreased the density of Oribatida when compared with the 40 m^3^ ha^− 1^ rate. The Oribatida presence dropped even further when slurries were combined with a bactericidal or fungicidal agent (used as slurry disinfectants). However, studies on oribatid mites in Mediterranean agricultural systems including crop management and fertilization practices are still lacking.

This study was set up in a Mediterranean agricultural area, under the umbrella of three mid-term fertilization experiments, which also differed in annual crop sequences. Twelve years after their establishment, experiments were sampled 3× along the following cropping season for oribatid mite identification. The ultimate aims of the work done were: (1) to characterize the response of oribatid mites to crop sequences and fertilization with animal droppings through some parameters, such as abundance, diversity index and number of individuals for each of the identified species, and (2) to evaluate the feasibility of the use of these oribatid parameters as indicators of potential soil degradation by agricultural management.

## Materials and methods

Three experiments were established in an experimental farm in the NE of Spain, and they were maintained for 12 years (2001–2013). They differed in the fertilization schedule (different organic or mineral fertilizers) and in the crop sequence. In the first two experiments, a rainfed winter cereal rotation of barley (*Hordeum vulgare* L.) and wheat (*Triticum aestivum* L.) was established. In the third experiment a maize (*Zea mays* L.) monoculture, under furrow irrigation, was maintained during the same period. In nine of 12 years, Bt-maize was used (it was also seeded in 2013). In the 2013–2014 cropping season, rape (*Brassica napus* var. *napus*) was introduced in the first experiment and wheat in the third. In this season, all crops were non-irrigated.

In this paper, the experiments are denominated according to the names of the crops included in the last 2-year rotation (until 2014 harvest): wheat–rape (experiment 1), wheat–barley (experiment 2), and maize–wheat (experiment 3).

### Site, soil and climate description

Experiments 1 and 2 were established in October 2001 (altitude 17 m above sea level, 42º03’15”N, 03º03’46”E). Experiment 3, due to the summer maize growing season, was established 5 months later, close to the other two experiments (altitude 18 m a.s.l., 42º03’02”N, 03º03’37”E). In all fields, the soil texture (0–0.3 m) is loamy. Illite is the dominant clay mineral with traces of kaolinite. The soils are non-saline, calcareous, and pH is 8.4. At the start of the experiments, soil organic carbon (0–0.3 m) varied from 8 to 10 g kg^− 1^ (Table S1). The soils were classified as Oxyaquic Xerofluvent (Soil Survey Staff 2022).

The area has a dry Mediterranean climate according to Papadakis classification (MAPA 1989). The annual average temperature is 15.8 °C with high summer temperatures (average 23 °C). The experimental year 2013–2014 showed an average temperature of 4–5 °C higher than in the historical period (Figure S1). The average annual precipitation is 649 mm.

### Historical management and fertilization practices in the three experiments

The general management for the three experiments is described in Table 1. In the 2012–13 harvest (8th July), the average biomass of winter wheat grain yields, in N-farmed plots, were close to 6 Mg ha^− 1^. In the 2013 maize harvest (13th September), the grain yield biomass in N-farmed plots was close to 12.5 Mg ha^− 1^.

**Table 1:** Agricultural broad management in the three experiments^a^

Experiment	Rotation (period)	Annual straw management (12-year rotation period)	Annual herbicide management^b^ (12-year rotation period)	Crop and sowing date (2013–2104 cropping season)
Wheat–rape	Barley–wheat (2001–2013)	Removed from fields. Stubble ploughed (~ 0.25 m). Last burial: October 14–15, 2013	At cereal post-emergence: 7.5% Bromoxinil + 7.5% Ioxinil (octanoic ester) + 37.5% Mecoprop (isooctyl ester and octanoic ester). Rate: 2 L ha^− 1^	Rape (October 29, 2013)
Wheat–barley				Barley (December 16, 2013)
Maize–wheat	Maize monoculture (2002–2013)	Straw ploughed (~ 0.25 m). Last burial: December 18, 2013.	Maize preemergence herbicide: Acetochlor 90% [2-chloro-N-(ethoxymethyl)-N-(2-ethyl-6-methylphenyl) acetamide]. Rate 1 L ha^− 1^ After maize emergence: Mesotrione 10% [2-(4-mesyl-2-nitrobenzoyl) cyclohexane-1,3-dione]. Rate: 1 L ha^− 1^	Wheat (December 23, 2013)

^a^ Foliar pesticides were not used. No herbicides or pesticides were applied to the rape

^b^ Herbicide schedule for cereals was maintained in 2013–2014

In wheat–rape, two annual fertilizer N treatments were studied (Table 2): (1) a control without N fertilization (0–0) and (2) treatment 47PS-0, using pig slurry (PS) before seeding at a wet-weight average rate of 47.3 Mg ha^− 1^ (Table 2). At pre-sowing, the control was complemented with 34.9 kg P ha^− 1^ as calcium superphosphate and 120.8 kg K ha^− 1^ as potassium sulphate, and 47PS-0 with 37.5 kg K ha^− 1^ as potassium sulphate because of the low K content of PS. The slurry was buried within 24 h from application, using a rotavator. No topdressing fertilization was done later during the cropping cycle.

**Table 2:** Annual average doses^a^ for a period of 12 cropping seasons (from 2001/2002 to 2013) of total-N, organic-N (Org-N), ammonium-N (NH_4_^+^-N) and organic matter (OM) applied with pig slurry (PS) or dairy cattle manure (CM) at presowing (Sw), and where mineral N (MN) was included later in the season as topdressing (TopD). Soil organic carbon (SOC, 0–10 cm depth) analyzed in July 2013 in experiments wheat–rape and wheat–barley, and in November 2012 in maize–wheat, are also included

Experiment	Treatments^b^	Total N	MN	Org-N	NH_4_^+^-N	OM	SOC^c^
(Period)	(Sw-TopD)			(kg ha^− 1^)		(Mg ha^− 1^)	(g C kg soil^− 1^)
Wheat–rape (2001–2013)	0–0	0	0	0	0	0	10.59b
	47PS-0	187 (161)	0	71 (53)	116 (108)	1.9 (2.1)	12.63a
Wheat–barley (2001–2013)	0–0	0	0	0	0	0	10.58b
	0-40MN	40	40	0	10	0	11.05b
	23CM-0	189 (110)	0	163 (98)	26 (12)	3.9 (2.5)	15.06a
Maize–wheat (2002–2013)	0–0	0	0	0	0	0	7.27c
	0-300MN	300 (0)	300 (0)	0	75	0	7.90c
	30CM-0	220 (0)	0	174 (0)	46 (0)	4.8 (0)	11.80b
	30CM-200MN	420 (0)	200 (0)	174 (0)	46 (0)	4.8 (0)	-
	60CM-0	440 (0)	0	348 (0)	92 (0)	9.6 (0)	14.63a

^a^ Numbers in parentheses are the applied amounts in the 2013–2014 cropping season (when they differ from the average for the previous 12 years). No disinfectant agents were added to the animal droppings

^b^ Treatment 0–0 is a control (no N applied); MN: mineral N fertilizer applied as calcium ammonium nitrate where the number refers to the amount of N applied as topdressing (kg N ha^− 1^); PS and CM: pig slurry or dairy cattle manure applied just before sowing at an average rate (wet-weight) of 47 Mg ha^− 1^ (PS) or 23, 30 or 60 Mg ha^− 1^ (CM)

^c^ Oxidizable organic carbon (Walkley-Black); for each experiment, mean values followed by different letters are significantly different (Duncan Multiple Range Test: *P* < 0.05)

In wheat–barley, three annual fertilizer N treatments were studied (Table 2): (1) a control without N fertilization (0–0), (2) treatment 0-40MN with mineral nitrogen (MN) fertilization at cereal tillering (end of February – mid-March), and (3) treatment 23CM-0 using dairy cattle manure (CM) at pre-sowing. Control (0–0) and MN fertilizer treatment (0-40MN) received at presowing 34.9 kg P ha^− 1^ as calcium superphosphate and 120.8 kg K ha^− 1^ as potassium sulphate. Furthermore, at cereal tillering, the 0-40MN treatment was complemented with 40 kg N ha^− 1^ as calcium ammonium nitrate. In treatment 23CM-0, manure was always applied before sowing at a rate that averaged (wet-weight rate) 22.5 Mg ha^− 1^ (Table 2). After application, CM was incorporated using a rotavator.

In maize–wheat, and for the period 2002–2013, five fertilization treatments (based on the source and dose of N) were applied annually (Table 2): (1) a control without N fertilization (0–0); (2) treatment 0-300MN with mineral nitrogen (MN), and (3) three treatments which include CM (30CM-0, 30CM-200MN, and 60CM-0). In maize, the CM doses were applied only before sowing (March). The CM was incorporated by a rotavator. The lowest CM dose, named 30CM-0 (30 Mg ha^− 1^), was complemented at sowing with 27 kg P ha^− 1^ (as calcium superphosphate) and 75 kg K ha^− 1^ (as potassium sulphate). In two treatments (0-300MN and 30CM-200MN), MN was applied (as calcium ammonium nitrate) in the stage of development of the Zadock maize V6-V8 (late May). The control and 0-300MN treatments received at presowing 55 kg P ha^− 1^ as calcium superphosphate and 150 kg K ha^− 1^ as potassium sulphate. In the 2013–2014 cropping season, no manure or mineral fertilizer was applied to wheat.

In all experiments, the rotavator tillage for seedbed preparation was used in a similar way and the rotavator operation was always extended to the entire surface occupied by the treatments.

Treatments were always distributed according to a randomized block design. Wheat–rape includes two plots per treatment, and wheat–barley and maize–wheat experiments include three plots per treatment. Plot sizes were 24 m^2^ for wheat–barley, and 30 m^2^ for wheat–rape and maize–wheat.

Before the last cropping season (2013–2014), plots were sampled (0–10 cm depth) for the organic carbon content of the soil to differentiate the changes associated with treatments (Table 2).

### Sampling, extraction, and taxonomic identification of mites

Throughout the 2013–2014 cropping season, all plots from the three experiments were sampled on three dates. The sampling dates for wheat–rape and wheat–barley were 7 October 2013, 12 March 2014, and 19 June 2014. The sampling dates for maize–wheat were 12 December 2013, 19 March 2014, and 2 July 2014. The first soil sampling was performed between 80 and 90 days after the harvest of the first crop (wheat or maize) and before the preparation of the seedbed for the second (rape, barley or wheat). The second soil sampling was performed before topdressing fertilization (if applicable) and the last soil sampling was performed just after the second crop harvest. Soil samples were taken at a depth of 0–0.05 m with soil cores (0.06 m in diameter with steel bores). For each plot, three soil cores were collected and a composite sample was obtained. Therefore, 84 composite samples were studied and statistically analyzed.

Within a 24-h period, oribatids were extracted with a modified Berlese-Tullgren funnel (Tullgren 1917) over a period of 7 days. The biota obtained were stored in ethanol at 70%. Adult oribatids were counted and identified at the species level using several taxonomic keys from Pérez-Íñigo (1993, 1997), Subías and Arillo (2001) and Weigmann (2006). The systematic ordination appearing in Subías (2022) was also followed with the exception of the species *Tectocepheus sarekensis* – the identification of which was additionally based on phylogenetic analyses according to Lauman et al. (2007) – and *Protoribates* (*P*.) *capucinus capucinus* and *Protoribates* (*Triaungius*) *obtusus*, which were included in the Haplozetidae family according to Walter and Latonas (2013). Mean abundance (individuals per m^2^) and the Shannon index of diversity *H*´ (Krebs 1999) were calculated. Differences in absolute abundance (individuals per soil volume sampled) between different species were also analyzed.

### Statistical analysis

Each experiment was analyzed separately. Data were analyzed using the SAS v.9.4 statistical package (SAS Institute 2014). Abundance and *H*’ data from the three samplings were analyzed using linear mixed models accounting for the repeated measures and the block structure (Littell et al. 1996). The MIXED procedure from the statistical package was chosen. The best model was selected using the Akaike information criterion (AIC; Akaike 1974), which is a widely used criterion that balances model fit and complexity. Specifically, we fitted linear mixed models with fertilization, time, and their interaction as fixed effects; block and its interactions, as well as plot, were considered random effects. In addition, different types of variance–covariance matrices were tested to model the correlation structure of data from the repeated measures performed. The significance of the fixed effects was tested using F-tests and the random effects were assessed using likelihood ratio tests. The homogeneity of variances and the normality of the distributions were also tested.

Number of individuals (absolute abundance of oribatids) for each species, after the harvest of the first crop (wheat in experiments 1 and 2, maize in experiment 3), were analyzed using a generalized mixed model with negative binomial distribution to control for overdispersion (Lord et al. 2012). Time was not included in this analysis. The GLIMMIX procedure from the statistical package was chosen. The best-performing model was selected using AIC. The model includes treatment, oribatid species and treatment × species interaction as fixed factors, and block as random effect to account for variability between blocks. We checked the goodness of fit of the model by examining the residuals and conducting a likelihood ratio test. We also calculated the overdispersion parameter to ensure that the negative binomial distribution was appropriate for the data.

In these analyses of species absolute abundance, if only one individual of an oribatid species was found (just in one plot of an experiment), these species were not included in the statistical analysis. Furthermore, in wheat–barley, oribatid species with an average total presence of < 1 individual per plot were also excluded.

## Results

### Oribatida total abundance and index of diversity

The maximum abundance of mites was always found at the first sampling (Table 3). A significant reduction with time was only found in wheat–barley. In wheat–rape and maize–wheat, differences with time depended on fertilization treatment.

**Table 3:** Average of oribatid abundance (individuals m^− 2^) for each of the three experiments and according to preceding crop (first name) and current crop (second name), fertilizer treatments^a^ and samplings^b^ throughout the current crop cycle. Marginal means (M. mean) are also included^c^

Experiment	Treatment	Sampling I	Sampling II	Sampling III	M. mean_treatment_
Wheat–rape	0–0	3832 b x	825 a x	766 a x	1808
	47PS-0	43,974 a x	1297 a z	13,734 a y	19,668
	M. mean_sampling_	23,903	1061	7250	
Wheat–barley	0–0	9038	1611	1690	4113
	0-40MN	9431	943	4559	4978
	23CM-0	12,339	786	4834	5986
	M. mean_sampling_	10,270 x	1113 y	3694 y	
Maize–wheat	0–0	1258 c x	39 a y	314 a y	537
	0-300MN	2083 b x	39 a x	707 a x	943
	30CM-0	2790 b x	157 a y	904 a y	1284
	60CM-0	4794 a x	236 a y	1140 a y	2057
	30CM-200MN	4048 a x	314 a y	786 a y	1716
	M. mean_sampling_	2994	157	770	

^a^ MN: mineral N fertilizer, applied at a rate of 40, 200 or 300 kg N ha^− 1^ yr^− 1^, according to the treatment in each experiment and applied as calcium ammonium nitrate (27% N) at topdressing; PS and CM: pig slurry and dairy cattle manure applied just before sowing at an average rate of 47.3 (PS, wheat–rape), 22.5 (CM, wheat–barley) and 30 or 60 Mg ha^− 1^ yr^− 1^ (CM, maize–wheat). In maize–wheat, it refers to fertilization applied annually during the previous 12 years, but not in the current cropping season

^b^ Sampling I: October for wheat–rape and wheat–barley, December for maize–wheat; sampling II: March; sampling III: June for wheat–rape and wheat–barley, July for maize–wheat

^c^ Means followed by different letters (*a* and *b* for differences between treatments, *x* and *y* for differences between samplings) are significantly different (least squares means: *P* < 0.05)

In wheat–rape and in October (sampling I), the abundance of mites in the PS fertilized plots (43,974 individuals m^− 2^) were more than ten times higher than in the control. Significant differences between both treatments disappeared from March (sampling II), but in March, PS attained its lowest value (1297 individuals m^− 2^). In wheat–barley, the reduction in abundance in March and June was 10× and 3.5×, respectively, when compared with figures in October (average of 10,270 individuals m^− 2^). In maize–wheat and in the first sampling, the control had the lowest abundance values (1258 individuals m^− 2^), numbers doubled for mineral and 30CM-0, and quadrupled for the rest of the treatments. In addition, with the exception of mineral treatment, abundance was reduced in March, after which the differences between fertilization treatments disappeared (Table 3).

The various fertilization treatments did not affect *H’* (Table 4). However, the diversity trend with time was different in each experiment. In wheat–rape, no differences were found. In wheat–barley, diversity declined with time, with higher values in October (1.07) than in June (0.59). In maize–wheat, the lowest diversity was recorded in March (0.24) whereas the first sampling did not differ (0.73) from the third (0.62).

**Table 4:** Means of Shannon index of diversity for each of the three experiments and according to preceding crop (first name) and current crop (second name), fertilizer treatments^a^ and samplings^b^ throughout the current crop cycle. Marginal means (M. mean) are also included^c^

Experiment	Treatment	Sampling I	Sampling II	Sampling III	M. mean_treatment_
Wheat–rape	0–0	1.57	0.66	1.43	1.22
	47PS-0	0.75	0.51	0.28	0.51
	M. mean_sampling_	1.16	0.56	0.85	
Wheat–barley	0–0	1.05	0.82	0.70	0.86
	0-40MN	1.19	1.04	0.64	0.96
	23CM-0	0.97	0.57	0.43	0.66
	M. mean_sampling_	1.07 x	0.81 xy	0.59 y	
Maize–wheat	0–0	0.86	0.00	0.40	0.42
	0-300MN	0.48	0.00	0.67	0.39
	30CM-0	0.83	0.32	0.31	0.45
	60CM-0	1.04	0.87	0.95	0.86
	30CM-200MN	0.46	0.40	0.75	0.54
	M. mean_sampling_	0.73 x	0.24 y	0.62 x	

^a^ MN: mineral N fertilizer, applied at a rate of 40, 200 or 300 kg N ha^− 1^ yr^− 1^ (according to the treatment in each experiment) as calcium ammonium nitrate (27% N) at topdressing; PS and CM: pig slurry and dairy cattle manure applied just before sowing at an average rate of 47.3 Mg ha^− 1^ yr^− 1^ (PS, wheat–rape), 22.5 Mg ha^− 1^ yr^− 1^ (CM, wheat–barley) and 30 or 60 Mg ha^− 1^ yr^− 1^ (CM, maize–wheat). In maize–wheat, it refers to fertilization applied annually during the previous 12 years, but not in the current cropping season

^b^ Sampling I: October for wheat–rape and wheat–barley; December for maize–wheat; sampling II: March; sampling III: June for wheat–rape and wheat–barley; July for maize–wheat

^c^ Means followed by different letters are significantly different (least squares means: *P* < 0.05)

### Absolute abundance of oribatid species

Eighteen oribatid mite species were found, belonging to 15 families (Table S2). The total number of species present in wheat–rape, wheat–barley, and maize–wheat was 11, 15, and 9, respectively.

In the first sampling and in the wheat–rape experiment, eight oribatid species were included in the statistical analysis (Table 5; Table S2): *Epilohmannia cylindrica cylindrica* (*E. cylindrica*), *Oribella pectinata*, *T. sarekensis*, *Scutovertex sculptus*, *Pseudotectoribates subsimilis subsimilis* (*P. subsimilis*), *Zetomimus* (*Protozetomimus*) *acutirostris* (*Z. acutirostris*), *Oribatula* (*Zygoribatula*) *excavata* (*O. excavata*) and *Galumna* (*G.*) *tarsipennata* (*G. tarsipennata*). An interaction (*P* = 0.0091) was found between species and fertilization treatments. In the control, a significant predominance of *Z. acutirostris* was found (Table 5; Table S2). No differences were detected among the other species. In the 47PS-0 treatment, *O. excavata* was the predominant species. *Zetomimus* (*Protozetomimus*) *acutirostris* was still important, but it only did not differ from *T. sarekensis* and *P. subsimilis* (Table 5; Table S2) and *P. subsimilis* did not differ from the rest of the species.

**Table 5:** Differences between species (associated probability) in the absolute abundance^a^ recorded in October in the wheat–rape experiment and according to the fertilization treatment

		*Oribella pectinata*	*Tectocepheus sarekensis*	*Scutovertex sculptus*	*Pseudotectoribates subsimilis*	*Zetomimus acutirostris*	*Oribatula excavata*	*Galumna* *tarsipennata*
Control (0–0)	*Epilohmannia cylindrica* (4)	0.5499 (6)	0.2549 (1)	0.7175 (3)	1.0 (4)	0.0083 (28)	0.1268 (11)	0.4016 (7)
	*O. pectinata*		0.1411	0.3596	0.5498	0.0111	0.3864	0.7897
	*T. sarekensis*			0.3731	0.2550	0.0136	0.0553	0.1115
	* S. sculptus*				0.7176	0.0079	0.0863	0.2592
	*P. subsimilis*					0.0083	0.1268	0.4016
	*Z. acutirostris*						0.0341	0.0135
	*O. excavata*							0.3810
Pig slurry	*E. cylindrica* (4)	− (0)	0.0372 (1)	1.0 (4)	0.0541 (38)	0.0259 (63)	0.0017 (584)	1.0 (4)
(47PS-0)^b^	*T. sarekensis*			0.0372	0.7647	0.7655	0.0208	0.0372
	* S. sculptus*				0.0541	0.0259	0.0017	1.0000
	*P. subsimilis*					0.5550	0.0142	0.0541
	*Z. acutirostris*						0.0309	0.0259
	*O. excavata*							0.0017

^a^ Numbers in parentheses correspond to the sum of the total number of individuals from all replicates (848 cm^− 3^, sampled at 5 cm depth)

^b^ During 12 cropping seasons and before the sampling, an average of 47.3 Mg ha^− 1^ yr^− 1^ of pig slurry was applied annually

In wheat–barley and first sampling, seven oribatid species were initially included in the statistical analysis. *Ceratozetes* (*C.*) *laticuspidatus* was present in one replicate of the 23CM-0 treatment (Table S2), the other eight values were ‘0’. Then the analysis was again carried out excluding this species (Table 6). Fertilization was not a significant variable (*P* = 0.053), only species was (*P* < 0.0001). *Oribatula* (*Z.*) *excavata* predominated, but the presence of *Z. acutirostris* was also higher than that of the rest of the species (Table 6; Table S2). The presence of *E. cylindrica* only overtook the presence of *T. sarekensis* (Table 6; Table S2). No differences were detected between the rest of the species analyzed: *P. subsimilis*, *G. tarsipennata* and *T. sarekensis.*

**Table 6:** Differences between species (associated probability) in the absolute abundance^a^ recorded in October in the wheat–barley experiment

Species	*Tectocepheus sarekensis*	*Pseudotectoribates subsimilis*	*Zetomimus acutirostris*	*Oribatula excavata*	*Galumna tarsipennata*
*Epilohmannia cylindrica* (40)	0.0181 (15)	0.1192 (22)	0.0177 (139)	< 0.0001 (510)	0.1198 (30)
*T. sarekensis*		0.3167	< 0.0001	< 0.0001	0.4156
*P. subsimilis*			0.0005	< 0.0001	0.8931
*Z. acutirostris*				0.0093	0.0010
*O. excavata*					< 0.0009

^a^ Numbers in parentheses correspond to the sum of individuals from all replicates (1272 cm^− 3^, sampled at 5 cm depth)

In maize–wheat, there was no interaction between the variables studied. No differences were identified between fertilization treatments. The presence of *O. excavata* was lower than that of *T. sarekensis* and *Acrotritia ardua americana* (*A. ardua*) (Table 7; Table S2).

**Table 7:** Differences between species (associated probability) in the absolute abundance^a^ recorded in December in the maize–wheat experiment

Species	*Tectocepheus sarekensis*	*Zetomimus acutirostris*	*Oribatula excavata*
*Acrotritia ardua* (130)	0.0717 (315)	0.9745 (24)	< 0.0001 (10)
*T. sarekensis*		0.9729	< 0.0001
*Z. acutirostris*			0.9829

^a^ Numbers in parentheses correspond to the sum of individuals from all replicates (1272 cm^− 3^, sampled at 5 cm depth)

## Discussion

### Oribatida abundance and index of diversity

The average abundance of oribatids (Table 3) was much lower than the average of 25,000 individuals m^− 2^ reported in rich agricultural soils from southeast USA (Coleman 2018), probably because of the constraints associated with a dry Mediterranean climate, such as low precipitation (Figure S1) and, consequently, the lower amount of available plant biomass produced, including roots. Nevertheless, our numbers are an indicator of the benefits of the use of animal droppings as fertilizer, as it allows to revert the decreasing trend in oribatid presence. Overall, the past application of slurries at the 47PS rate (wheat–rape) or manures at 23CM (wheat–barley) or from 30CM to 60CM (maize–wheat) resulted in an increase in soil organic carbon (Table 2). The latter, in wheat–rape or maize–wheat first sampling coincides with the trend of mite abundance increase, when it is compared with the control or with mineral fertilization (Table 3). In fact, George et al. (2017) described a positive relationship between oribatid abundance and total organic carbon.

The maximum abundance values before sowing (Table 3) could be explained by management. The first sampling was done before the physical disturbance associated with seedbed preparation (Usher et al. 1982) and cultivation is known to reduce the presence of soil arthropods (Edwards and Lofty 1975). The previous positive effect of organic fertilization on abundance was not further recorded, probably because it was counteracted by tillage, which favors initial soil compaction (Mateo-Marín et al. 2021). Arroyo et al. (2013) described a negative correlation between oribatid mite abundance and soil bulk density. In fact, Oribatida are generally associated with the 6–90 μm pore size class of soil (Vreeken-Buijs et al. 1998), and mesoporosity (diameters between 30 and 75 μm) and macroporosity (diameters > 75 μm) are the most sensitive to management practices (Yu et al. 2018). Furthermore, mites are not capable of creating habitable pores in soil (Porre et al. 2016). The observed relatively slow recovery of the oribatid population also agrees with the dynamics described by Zaitsev et al. (2002).

Average abundances in the different experiments alert about some additional negative disturbances in maize–wheat (Table 3), as it is considered that oribatid populations rapidly decline when their habitat is damaged (Siepel 1996). Early warning arises when looking at the abundance values of the controls from the various experiments, or when a similar dose of organic matter was added (30CM in maize–wheat vs. 23CM in wheat–barley; Tables 2 and 3) or when similar soil organic carbon was present (60CM in maize–wheat vs. 23CM in wheat–barley; Table 2). Although the three experiments were analyzed separately, the overall lowest abundance figures in maize–wheat might be related to previous maize crop management, including the use of Bt-maize and the specific pesticide schedule. Oribatids feed on soil microflora and decomposing plants which contain the toxin. However, Zwahlen et al. (2007) did not find major changes in the decomposition of Bt-maize residue and in the composition of the soil organism community. Arias-Martín et al. (2016) also suggested that soil microarthropods are not negatively affected after 3 years of Bt-maize cultivation. Nevertheless, Tapp and Stotzky (1998) stated that the amount of retention of toxin varies according to clay mineral composition (higher in montmorillonite than in kaolinite) and pH (higher in soils with low pH). In our soils – with high pH and low clay (illite/kaolinite) content (68 g kg^− 1^, Table S1) – the persistence of insecticide activity might be low. However, initial abundance differences between fertilization treatments after maize might be related to the organic matter added annually (Table 2) that could counterbalance the immediate impact of the toxin by binding a fraction of it (Saxena and Stotzky 2001). On the other hand, the herbicide acetochlor used in maize shows genotoxicity (Wilson et al. 1996). However, its weakly polar compounds can show remarkable adsorption on soils where the organic content is low (Lengyel and Földényi 2003). As the organic matter content of soils plays an important role in adsorption, it helps to explain (in sampling I) the increase in abundance of oribatids as the doses of CM (applied in previous years) increased (Tables 2 and 3).

It is argued that the *H*´ index is unspecific as many factors influence diversity (van Straalen 1998). In our study, the evolution of diversity with time has different patterns, although the tendency to higher *H*´ values in the autumn (Table 4) coincides with the findings of Arroyo and Iturrondobeitia (2006) in pastureland or forest soil in NW Spain. In wheat–barley, a significant decrease in diversity was observed as the cropping season advanced. Temporal diversity dynamics of the soil mesofauna across sampling months are common, as mesofauna is sensitive to changes in climatic factors (Wu and Wang 2019). Furthermore, in maize–wheat, the reduction in diversity in March sampling could be partially linked to the initial low number of species in this experiment (Table S2) and the period with low precipitation after seedbed preparation in December (Fig. S1). Furthermore, as 2013–2014 was a residual fertilizer cropping season, no benefits of annual organic application can be added.

### Absolute abundance of oribatid species

Although 18 species of oribatids are recorded in the study (Table S2), clearly at most half of them are the focus, as some of the described species (e.g., *Passalozetes africanus*, *Lucoppia burrowsi*, *Dometorina* sp., *Berlesezetes ornatissimus mirus*) are serendipitous rarities. The low number of species found is a characteristic of agricultural systems (Curry 1986). Drawing an analogy with litter, the richness of oribatids declines with decreased litter richness (Hansen and Coleman 1998). Consequently, after 12 years of maize monoculture, it is quite likely this will lead to a further decrease in the number of species present (Table S2), when compared with the two-crop rotation system.

In the fertilized wheat–rape plots and in the wheat–barley experiment, *O. excavata* predominated, followed by the presence of *Z. acutirostris* (Tables 5 and 6; Table S2). The reduction in the presence of *O. excavata* after seedbed preparation could be partially explained because it is affected by conventional tillage (Franchini and Rockett 1996). Its presence recovered as the crop developed and as the roots grew, but mainly when organic matter, from animal droppings, was also buried (Table S2). The presence of O. *excavata* has been reported to be lower at high N rates (Bosch-Serra et al. 2014). Thus, its prevalent presence in wheat–rape and wheat–barley will indicate that the historical average of applied N rates (187–189 kg N ha^− 1^) or in the current season (161–110 kg N ha^− 1^) with PS or CM, respectively (Table 2), should not be considered disturbing. However, the latter context does not explain O. *excavata* low presence in maize–wheat.

Aoki (1979) lists Ceratozetidae as being a family insensitive to environmental impact, but he placed the family in the intermediate group. Although this may not be true for all species of ceratozetids, it agrees with the presence of *Z. acutirostris* in our experiments, although it was not always dominant (Tables 5, 6 and 7; Table S2).

In relation to the presence of *T. sarekensis* in all experiments (Table S2), it is noticeable that the Tectocepheidae family is a primary colonizer in agricultural soils (Behan-Pelletier 1999). The species *T. sarekensis* is an opportunistic herbofungivore and is widely distributed in different European and North American habitats (Coleman et al. 2018). It is encountered in cultivated plots (Rockett 1986), but also at highly metal-contaminated sites (Feketeová et al. 2021; Skubala et al. 2012).

The presence of other specimens of *Epilohmannia* sp. and *Galumna* sp. is less frequent in cultivated soils than in grassy or woody areas (Rockett 2009). In fact, *E. cylindrica* is sensitive to soil disturbances, as its presence is reduced by conventional tillage when compared to minimum tillage (Bosch-Serra et al. 2014). These species even disappear in maize–wheat (Table S2), although it has been described they are able to adapt to a wide range of habitats, including urban environments (Shevchenko and Kolodochka 2013).

*Scutovertex sculptus* disappeared after the first sampling (Table S2). According to Smrž (2002), the *Scutovertex* genera survive in an inactive state in the presence of drought, for a period that can last up to 20 days from which they can recover. This fact underscores the potential influence of the scarce winter rainfall (Figure S1) on the reduction of species diversity in March sampling (Table S2).

The presence of the *Oppiella* genera (mostly thelytokous) should be considered a serendipitous rarity (Appendix S2), which contrasts with the fact that it has also been described as characteristic of disturbed soils in cultivated sites (Norton and Silman, 1985). In fact, the presence of the fungivorous grazer *Oppiella nova nova* is residual, although it can survive in compacted soils (Franchini and Rockett 1996). Its reduced presence is a matter of concern, as it has been described along with *T. sarekensis* as suitable for characterizing degraded ecosystems along an input gradient (Gulvik 2007) in arable files, pasture, and fallow. However, its sensitivity to drought (Siepel 1996) might have controlled its presence in our experiments.

In the first sampling, more detailed differences were found. In wheat–rape, the application of the slurry mainly improved the presence of *O. excavata*, followed by *Z. acutirostris*, *T. sarekensis*, and *P. subsimilis* (Table 5; Table S2). The increase of *O. excavata* abundance in fields receiving pig slurries coincides with previous findings of Bosch-Serra et al. (2014). The species *E. cylindrica* and *Scutovertex sculptus* remained unaffected to such practice, probably because *E. cylindrica* is present in cereal systems regardless of the fertilizer origin (Arroyo et al. 2005), and most of the common habitats for the eurytopic *Scutovertex sculptus* are less intensively managed environments, such as non-cultivated (Iturrondobeitia et al. 2004) or abandoned croplands (Arroyo et al. 2005).

In maize–wheat, the presence of *T. sarekensis* and *Z. acutirostris vs. O. excavata* was noticeable. *Zetomimus* (*P.*) *acutirostris* showed an irregular presence, being found mainly in the 60CM treatment and not present in the 300MN and 30CM-200MN treatments (Table 7, Table S2). Previous fertilization cannot explain the significant presence of the two main species. The Tectocepheidae has been classified as the most insensitive group to environmental destruction (Rockett 1986) or strong disturbances (Zaitsev et al. 2002), Feketeová et al. (2021) describe *Z. acutirostris* as a suitable indicator of improper human intervention in an ecosystem. As an example, it has been described that under stress induced by organophosphate or chlorinated hydrocarbon products, its presence was enhanced (Behan-Pelletier 1999). In our case, all maize–wheat plots were previously treated with the same products, in particular with the herbicide acetochlor. A chronic exposure value to acetochlor has been established for mice (USEPA 2006). However, it is unknown whether its presence in the soil system might affect the oribatid numbers, mainly when considering that one or two generations per year is the common pattern, and also because females do not lay many eggs. In fact, *O. excavata* with a sexual reproductive mode almost disappeared, whereas it was one of the most abundant species in the other experiments (Table S2). Our results on the prevalence of two parthenogens seem to contradict Maraun et al. (2020), who found that the high frequency of parthenogenesis is not associated with a low density of oribatid mites. The key point is that in their study they did not have densities below 5000 individuals m^− 2^ as we mainly found in maize–wheat. Furthermore, there were no resource limitations that favour sexual reproduction (Seniczak et al. 2016). In our context, where oribatid mite densities are low and sperm transfer is more difficult, *T. sarekensis* and *A. ardua* have an advantage because they reproduce via parthenogenesis (Pachl et al. 2021). This characteristic can also explain their prevalence after a Bt-maize cropping season. In fact, Siepel (1995) associates thelytokous reproduction with permanent and persistent pollution. Our results also agree with Owojori et al. (2012) who found that reproduction (of *Oppia nitens*) was a more sensitive endpoint to toxicity (caused by the organic chemical benzo[α]pyrene and metals) than to mite survival. In general, the prevalence of parthenogens may be an indicator of the (high) death rate among mites due to an important disturbance related to maize monoculture and the associated management.

Although it is well know that oribatid mites have low diversity in arable fields (Bosch-Serra et al. 2014), our results sustain the eco-scheme concept of the EU new Common Agricultural Policy (European Commission 2020). In our work, two-crop rotation (in front of maize monoculture) and the use of animal droppings as fertilizer favour the presence of multiple Oribatida species involved in the decomposition of plant litter and other organic material. This function is one of the aspects of the circular nutrient economy and carbon sequestration. Furthermore, the predominance of some specific thelytokous species (in maize monoculture) might be used as an early warning of potential environmental degradation in dry Mediterranean areas linked to agricultural practices, despite the maintenance or even the increase of soil organic carbon. Further research should support the extension of such indicators to different agricultural systems.

## Electronic Supplementary Material

Below is the link to the electronic supplementary material.

Supplementary Material 1

## Data Availability

The datasets generated during and/or analyzed during the current study are available from the corresponding author on reasonable request.
