# Supplementary material for: Oribatid mites in different Mediterranean crop rotations fertilized with animal droppings
Source: Exp Appl Acarol. 2023 Jun 20;90(3-4):185–202. doi: 10.1007/s10493-023-00812-4 (PMC10406719; doi:10.1007/s10493-023-00812-4)
Supplement: Supplementary file 1 — Supplementary Material 1 [file 10493_2023_812_MOESM1_ESM.docx]

**Supplementary Information**

| 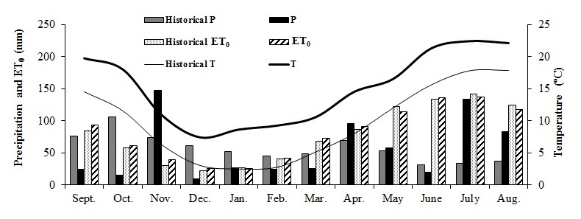 |
| --- |
| **Figure S1** Mean monthly precipitation (P), air mean temperature (T) and reference evapotranspiration (ET_0_) according to Penman-Monteith equation (Allen et al. 1998) for the historic period (1993–2021) and for the experimental period (September 2013–August 2014) |

**Table S1** Soil characteristics^a^ (0-30 cm depth) in the three experiments: wheat–rape, wheat–barley and maize–wheat

| Parameters | Wheat–rape and wheat–barley | Maize–wheat |
| --- | --- | --- |
| Sand^2^ (2000 > Ø > 50 µm) (g kg^-1^) | 458 | 497 |
| Silt^2^ (50 > Ø > 2 µm) (g kg^-1^) | 413 | 435 |
| Clay^2^ (Ø < 2 µm) (g kg^-1^) | 129 | 68 |
| pH (soil:distilled water, 1:2.5) | 8.4 | 8.4 |
| Electrical conductivity (1:5; dS m^-1^, 25 ºC) | 0.13 | 0.19 |
| Soil organic carbon (g kg^-1^; Walkley-Black) | 10.0 | 7.6 |
| Calcium carbonate equivalent (g kg^-1^) | 129 | 140 |

^a^ Composite samples (0–30 cm) were obtained at the beginning of each fertilization experiment, 12 years before the first mite sampling in the 2013–2014 cropping season

^2^Ø: particle apparent diameter

**Table S2** List of oribatid families and species. Total number of individuals^a^ for each of the three experiments according to preceding crop (first name) and current crop (second name), fertilizer treatments^b^ and for the three samplings in the 2013–2014 cropping season (at presowing of the second crop/ at March/ after second crop harvest) are included

|  | | Wheat–rape | | Wheat–barley | | | Maize–wheat | | | | |
| --- | --- | --- | --- | --- | --- | --- | --- | --- | --- | --- | --- |
| Family | Species | 0-0 | 47PS-0 | 0-0 | 0-40MN | 23CM-0 | 0-0 | 0-300MN | 30CM-0 | 60CM-0 | 30CM-200MN |
| Epilohmanniidae | *Epilohmannia cylindrica cylindrica* | 4/ 3 / 3 | 4/ 4 / - | 16/ 4 / - | 13/ 3/ - | 11/ 4/ - | - | - | - | 1/ -/ - | - |
| Euphthiracaridae | *Acrotritia ardua americana* | -/ 2/ 1 | -/ -/ 2 | 2/ -/ - | 3/ -/ 1 | - | 9/ -/ - | 22/ 1/ 2 | 29/ 2/ 2 | 33/ 2/ 1 | 37/ 5/ 1 |
| Oribellidae | *Oribella pectinata* | 6/ -/ - | - | - | - | 2/ -/ - | - | - | - | - | - |
| Oppiidae | *Oppiella nova nova* | 1/ -/ - | -/ 1/ - | - | - | - | 1/ -/ - | - | - | - | - |
| Tectocepheidae | *Tectocepheus sarekensis*^c^ | 1/ -/ 1 | 49/ 1/ 8 | 5/ 1/ - | 9/ -/ 21 | 1/ -/ - | 20/ 1/ 1 | 30/ -/ 3 | 36/ 1/ 8 | 65/ 2/ 11 | 64/ 2/ 11 |
| Scutoverticidae | *Scutovertex sculptus* | 3/ -/ 1 | 4/ -/ - | - | 4/ -/ - | 3/ -/ - | - | - | - | - | - |
| Passalozetidae | *Passalozetes* (*P.*) *africanus* | -/ -/ 1 | - | - | - | - | - | - | - | - | - |
| Microzetidae | *Berlesezetes ornatissimus mirus* | - | - | - | 1/ -/ - | 4/ -/ - | - | - | - | - | - |
| Tegoribatidae | *Pseudotectoribates subsimilis subsimilis* | 4/ -/ 1 | 38/ -/ 2 | 4/ -/ 3 | 14/ -/ 1 | 4/ -/ 1 | - | - | - | - | - |
| Ceratozetidae | *Ceratozetes* (*C.*) *laticuspidatus* | - | - | - | - | 10/ -/ - | - | - | - | - | 4-/ -/ - |
|  | *Zetomimus* (*Protozetomimus*) *acutirostris* | 28/ 2/ 4 | 63/ 1/ - | 48/ 4/ 9 | 43/ 9/ 13 | 48/ 3/ 14 | 1/ -/ 6 | / -/ 13 | 2/ 1/ 13 | 21/ 2/ 14 | -/ -/ 8 |
| Oribatulidae | *Lucoppia burrowsi* | - | - | -/ -/ 2 | - | - | - | - | - | - | - |
|  | *Oribatula* (*Zygoribatula*) *excavata* | 11/ 6/ 1 | 584/ 15/ 221 | 151/ 31/ 29 | 130/ 10/ 80 | 229/ 13/ 108 | 1/ -/ - | 1/ -/ - | 4/ -/ - | 2/ -/ 3 | 2/ -/ - |
| Hemileiidae | *Dometorina* sp. | - | - | - | 2/ -/ - | 1/ -/ - | -/ -/ 1 | - | - | - | - |
| Scheloribatidae | *Scheloribates* sp. | - | - | - | - | - | - | - | - | - | -/ 1/ - |
| Haplozetidae^d^ | *Protoribates* (*P.*) *capucinus capucinus* | - | - | 2/ -/ - | -/ 1/ - | - | - | - | - | - | - |
|  | *Protoribates* (*Triaungius*) *obtusus* | - | - | 2/ 1/ - | - | - | - | - | - | - | - |
| Galumnidae | *Galumna* (*G.*) *tarsipennata* | 7/ 1/ - | 4/ -/ - | 8/ -/ - | 21/ 1/ - | 1/ -/ - | - | - | - | - | - |

^a^ Number of individuals in 848 cm^-3^ in wheat–rape and in 1272 cm^-3^ in wheat–barley and maize–wheat; all sampled at 5 cm depth. The hyphen (-) was used when any mite, belonging to a specific species, was found in a specific sampling or in all samplings.

^b^ MN: mineral N fertilizer, applied at a rate of 40, 200 or 300 kg N ha^-1^ yr^-1^ (according to the treatment in each experiment) as calcium ammonium nitrate (27%) at topdressing; PS and CM: pig slurry and dairy cattle manure applied just before sowing at an average rate of 47.3 (PS, wheat–rape), 22.5 (CM, wheat–barley) and 30 or 60 Mg ha^-1^ yr^-1^ (CM, maize–wheat). In maize–wheat, it refers to fertilization applied annually during the previous 12 years, but not in the current cropping season.

^c^ This species is morphologically identified as *Tectocepheus velatus sarekensis* (Subías, 2022) but according to Lauman et al. (2007) it is considered a distinct species: *Tectocepheus sarekensis.*

^d^ These species were included in the Haplozetidae family according to Walter and Latonas (2013).
